# Effects of pseudoexperience on the understanding of hemiplegic movements in physical therapists: An fMRI study

**DOI:** 10.1016/j.nicl.2019.101845

**Published:** 2019-04-30

**Authors:** Rui Watanabe, Narumi Katsuyama, Nobuo Usui, Masato Taira

**Affiliations:** Department of Cognitive Neurobiology, Graduate School of Medical and Dental Sciences, Tokyo Medical and Dental University, 1-5-45 MD Tower 7F Yusima, Bunkyo-ku, Tokyo 113-8549, Japan

**Keywords:** Action observation, Hemiplegia, Mentalizing, Physical therapist, Temporoparietal junction, NPs, naïve participants, HHM, hemiplegic hand movements, QMF, a questionnaire regarding movement-associated feeling states

## Abstract

Physical therapists (PTs) are required to obtain an accurate understanding of the physical and mental states of their patients through observational assessment. To perform comprehensive observational assessments of patients' movements, PTs likely need to engage their own neural systems involved in action understanding and theory of mind, such as the action observation network (AON) and the right temporoparietal junction (rTPJ). Both systems are modulated by the observer's actual experience with the observed movements. Although, most PTs do not have physical experience with neurological disabilities, they routinely examine hemiplegic movements in stroke patients, and are thus considered to have acquired pseudoexperience with hemiplegia. We hypothesized that the PTs' pseudoexperience with hemiplegia would modulate the neural system associated with the understanding of others to elaborately comprehend the physical and mental states associated with hemiplegia. To investigate our hypothesis, we recruited 19 PTs and 19 naïve participants (NPs) to undergo functional MRI (fMRI) for cortical activity measurement while viewing videos of hemiplegic (HHM) and non-hemiplegic (non-HHM) hand movements. The participants subsequently viewed the same videos again outside the MRI scanner, and evaluated the observed hand movements via a questionnaire. Compared to the NPs, the PTs showed greater activation in the AON and rTPJ while observing HHMs. Psychophysiological interaction analyses revealed increased connectivity between the rTPJ and AON when the PTs viewed the HHMs. Behavioral analyses further indicated that the PTs more accurately assessed feeling states associated with HHMs than did NPs. These findings suggest that the PTs' pseudoexperience modulates the AON and rTPJ, enabling them to better understand hemiplegia-associated feeling states.

## Highlights

• We tested the effect of pseudoexperience on the understanding of hemiplegia
• Physical therapists (PTs) or naïve participants observed hemiplegic movements (HHMs)
• The action observation network and temporoparietal junction were more active in PTs
• PTs provided higher subjective ratings regarding their feelings associated with HHM
• The accumulated pseudoexperiences of PTs may enable them to better understand HHM

## 1 Introduction

In physical therapy, observational assessment of movements in patients with disabilities, as well as examination by touch, has been believed to be an essential method for appropriate treatment (Bobath, 1990; Schenkman et al., 2006). Through such an assessment, physical therapists (PTs) are required to accurately identify the physical and mental states of their patients, which includes movement difficulty, muscle stiffness, and negative affective feeling (Bernhardt et al., 1998; Raine et al., 2009). During observational assessments of patient movements, PTs likely need to engage their own neural systems involved in action understanding and theory of mind, such as the action observation network (AON) and right temporoparietal junction (rTPJ), to obtain an accurate understanding of the bodily and mental states of the patient.

The action observation network (AON)—which encompasses the mirror neuron system (MNS), posterior superior temporal sulcus, somatosensory areas, and motion-related visual areas—plays a substantial role in mapping observed actions onto the observer's corresponding motor representation. Thus, it is thought that the AON response to observed actions enables functional comprehension of actions performed by others (Caspers et al., 2010; Cross et al., 2006; Gallese et al., 2004; Kana and Travers, 2012; Keysers et al., 2010; Rizzolatti and Craighero, 2004).

The right temporoparietal junction (rTPJ) is engaged during action observation when the observer perceives the action-related mental state or takes the perspective of the action performer (Bernhardt and Singer, 2012; Blanke and Arzy, 2005; Blanke et al., 2005; Corbetta et al., 2008; Decety and Lamm, 2007; Mizuguchi et al., 2016; Saxe, 2006; Wurm and Schubotz, 2018). The rTPJ is one of the neural substrates comprising the theory-of-mind (ToM) network. Prior data suggest that the rTPJ preferentially responds to understanding another person's affective and cognitive mental state as opposed to perceiving the kinematics of observed action (Wurm et al., 2011; Wurm and Schubotz, 2018). Notably, Mizuguchi et al. (2016) revealed that when subjects observed another person's action, the rTPJ was closely associated with perceiving the other person's feeling of effort for that action.

Engagement of these neural networks is likely modulated by the observer's actual physical experiences and familiarity with the observed movements (Aziz-Zadeh et al., 2012; Calvo-Merino et al., 2006; Cross et al., 2009, Cross et al., 2012; Liew et al., 2013; Pilgramm et al., 2010). The AON is recruited during the observation of movements that the observer has previously experienced, enabling the observer to reference stored representations when attempting to understand those observed movements (Calvo-Merino et al., 2006). Some data suggest that the AON is also activated during the observation of movements that healthy individuals cannot experience, such as robotic movements (Cross et al., 2012; Gazzola et al., 2007; Liew et al., 2013; Shimada, 2010). This suggests that the observation of impossible movements requires paying attention and that motor representations are activated to interpret the sensation of observing unexperienced movements. However, a study by Cross et al. (2009), which controlled for the experience factor, clearly revealed that actual experiences have a closer link with AON recruitment when it comes to understanding observed movements when compared to no physical experiences. In the case of activation patterns of the rTPJ, Cheng et al. (2007) reported that physicians with acupuncture expertise exhibit greater rTPJ activation while observing acupuncture treatment compared to non-physician participants without acupuncture expertise. They concluded that based on their practical experiences, physicians were physically aware that acupuncture situations would be painful, possibly leading them to automatically observe the situation from an external viewpoint. In other words, an observer's physical familiarity, attributable to treatment experiences, may promote increased neural responses in the rTPJ.

Although, most PTs have not physically experienced severe neurological diseases, such as stroke, skilled PTs correctly identify the physical and mental states of patients with hemiplegia to some extent. PTs have abundant treatment and examination experience for patients with hemiplegia through their somatosensory and visual systems mutually (e.g., touching and observation of patients with hemiplegia). Thus, they are considered to have acquired pseudoexperience with hemiplegic movements attributable to their clinical processes. Therefore, we hypothesized that the PTs' acquired pseudoexperience with hemiplegia would effectively produce enhanced brain activation in the neural system associated with understanding others (i.e., AON and rTPJ) and enable them to sophisticatedly comprehend the physical and mental states associated with the hemiplegic movements.

In the present study, we aimed to investigate our hypothesis, and recruited highly-skilled PTs who were experienced in hemiplegia treatment and naïve participants (NPs) who were not PTs and had no prior experience with hemiplegia. We then measured blood-oxygen-level dependent (BOLD) signals using functional MRI (fMRI) while the participants viewed movies of hemiplegic hand movements performed by individuals with stroke-induced hemiplegia. The participants observed hand movements performed by the hemiplegic and non-hemiplegic hands in two different conditions.

We also developed a detailed questionnaire to assess the degree of understanding of hemiplegic movements. Other than pain-related investigations, few previous fMRI studies of action observation have effectively utilized subjective rating scales. However, such measures are required to confirm whether the observers actually understand the physical states associated with the observed movements. Here we utilized a detailed questionnaire regarding the bodily and mental states associated with hemiplegic movements, based on common subjective feeling states arising from the hand movements of the six individuals with hemiplegia depicted in the video stimuli.

In addition to this questionnaire, we adopted another questionnaire that examines the degree of an individual's empathic trait, the interpersonal reactivity index (IRI) (Davis, 1983). Previous studies have suggested that individuals with higher empathic traits exhibit more enhanced activation in brain systems associated with understanding and empathizing with others (Lamm et al., 2010; Myers et al., 2013; Moriguchi et al., 2007). We hypothesized that rather than highly empathic traits, pseudoexperience with hemiplegia should preferentially play a role in activating such brain systems and understanding the feeling states associated with hemiplegia. To test this hypothesis, we asked the participants to complete the IRI questionnaire to measure their empathic traits after completion of the former questionnaire.

## 2 Materials and methods

### 2.1 Participants

We recruited 38 participants (23 women and 15 men; mean age, 30.9 ± 4.2 years; range, 24–39 years) who had no history of neurological or psychiatric illnesses, and who were all right-handed according to the Edinburgh Handedness Inventory (Oldfield, 1971). All the participants were college graduates. Nineteen participants (11 women and 8 men; mean age, 32.4 ± 3.7 years) were physical therapists (PTs) who had at least 5 years of clinical experience with treating patients with hemiplegia (mean years of experience, 8.8 ± 2.3), all of whom had consistent daily contact with patients with hemiplegia. The remaining 19 participants (12 women and 7 men; mean age, 29.4 ± 4.3 years) were naïve participants (NPs) who had little to no experience examining or observing patients with hemiplegia. After the experiment, we confirmed that they had never made complete contact with individuals with hemiplegia. All participants provided written informed consent. The study protocol was approved by the Institutional Ethics Committee of Tokyo Medical and Dental University (Approval Number: 1264) and was conducted in accordance with the Declaration of Helsinki.

### 2.2 Stimuli

**Fig. 1:**
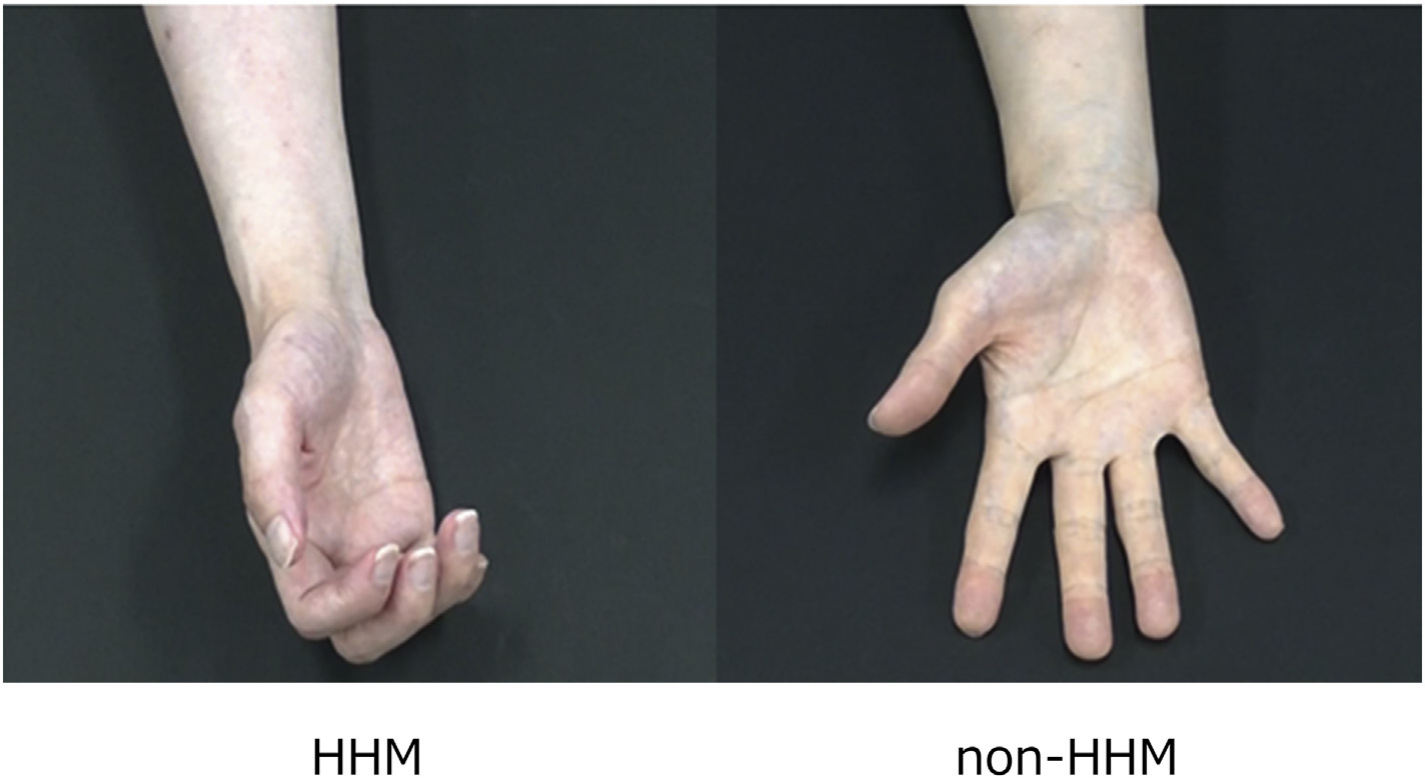
Two kinds of video clips were presented to participants. Left: The hemiplegic hand movements (HHM) condition. Right: The non-hemiplegic hand movements (non-HHM) condition.

The stimuli comprised color video clips depicting individuals with hemiplegia performing clasping–unclasping motions with their hands. Recordings were made using a digital video camera (GZ-RX500-B; JVC, Inc., Kanagawa, Japan), and included both hemiplegic hand movements (HHMs) and non-hemiplegic hand movements (non-HHMs) (Fig. 1) performed by six individuals with stroke-induced hemiplegia (three women and three men). Three individuals had right-sided hemiplegia, and the other three had left-sided hemiplegia. All had similar degrees of hemiplegia (mean Motor Status Scale hand score, 5.7 ± 1.2), such that they were capable of incomplete active flexion and extension, only with all of their fingers in synergy (Ferraro et al., 2002). None had orthopedic impairments in the hemiplegic hand or functional impairments in the non-hemiplegic hand.

In the present study, all of the stimuli were presented from the third person perspective (3PP), as PTs generally observe and assess their patients from the 3PP. Additionally, in many previous studies investigating imitative behavior or action observation, the 3PP has been considered to be another person's viewpoint (as if the observers are facing the others) (Bortoletto et al., 2013; Jackson et al., 2006; Kelly and Wheaton, 2013; Vistoli et al., 2016; Watanabe et al., 2017). Based on the fact that PTs observe the patients as “other”, we considered that the 3PP was more appropriate for our study compared to the first person perspective, in which the observers observe movement from their own perspective.

To create additional stimuli, each video clip was flipped in the horizontal direction. For example, we recorded a right-sided HHM and a left-sided non-HHM from one individual with right-side hemiplegia. We then inverted the video clips to create a left-sided HHM and a right-sided non-HHM. Thus, four video clips were created from each individual (two HHMs and two non-HHMs), yielding a total of 24 video clips (12 HHMs and 12 non-HHMs) from the six individuals with hemiplegia. Each video clip included one clasping–unclasping movement, and lasted 8 s. Immediately after recording the video clip, the individuals with hemiplegia were asked to give a free response to the following question: “What did you feel regarding your hand and the hand movement while performing the clasping–unclasping movement?” Based on their responses, we created a questionnaire regarding movement-associated feeling states (QMF) to assess the study participants' understanding of observed actions (see Post-scanning ratings). The participants were not provided information about the individuals performing the hand movements to ensure that they were not biased toward the video stimuli during the present experiment.

### 2.3 fMRI procedure

For each observer, the experimental session included the presentation of both HHM and non-HHM video clips, with 12 hemiplegic trials and 12 non-hemiplegic trials. Each trial lasted 18 s, and involved the presentation of a white fixation cross for 1 s, followed by an 8-s video clip, another white fixation cross for 1 s, and repetition of the same 8-s video clip; thus, the same video clip was presented twice in each trial. The hemiplegic and non-hemiplegic trials were alternated in a fixed sequence, with an 18-s inter-trial interval, during which a white fixation cross was presented on a black background. A behavioral pilot study indicated that the participants may receive a more intense impression if the video is shown twice. Thus, we repeatedly showed the same video in one trial to our study participants. In both trials, the video clips were presented in random order. The total fMRI session duration was 14 min 24 s. All video clips were presented using Presentation 18.2 (Neurobehavioral Systems, Inc., Albany, CA, USA).

In the scanner, participants were asked to observe the hand movements and to think about what the performers were physically and mentally feeling regarding their hands during each movement. They were also informed that they would be asked the same question after observing the same video clips outside of the scanner.

### 2.4 Imaging data acquisition

MRI was performed using a 3 T whole-body superconducting scanner system (MAGNETOM Spectra 3 T; Siemens, Inc., Germany) equipped with a quadrature detection birdcage head coil and an actively shielded gradient coil. Functional scans were performed using T2*-weighted echo-planar imaging (EPI) gradient-echo sequences. The BOLD-sensitive single-shot EPI sequence parameters included a repetition time (TR) of 2000 ms, echo time (TE) of 30 ms, 77° flip angle (FA), 64 × 64 matrix size, 192 × 192 mm^2^ field-of-view (FOV), slice number of 34, slice thickness of 3.0 mm, and a gap between slices of 0.75 mm. For each participant, we also acquired a T1-weighted anatomical image using the following parameters: TR = 1900 ms, TE = 2.42 ms, FA = 9°, FOV = 250 mm, 256 × 256 matrix size, and slice thickness of 1 mm.

### 2.5 Post-scanning ratings

Immediately following the fMRI scan, participants were asked to view each video clip again on a laptop computer, and to use the QMF to answer the question “What does the performer feel regarding the hand and hand movement while performing the clasping–unclasping movement?”

The QMF contained the following 11 negative and positive items (with the former item being negative and the latter positive): (1) the hand is heavy – the hand is light (HEAVY); (2) the hand is stiff – the hand is soft (STIFF); (3) the hand is rigid – the hand is flexible (RIGID); (4) I cannot relax the hand – I can relax the hand (NOT RELAX); (5) the movement is awkward – the movement is smooth (AWKWARD); (6) my grip is strong – my grip is weak (STRONG GRIP); (7) I cannot control the movement as I thought – I can control the movement as I thought (NO CONTROL); (8) the movement is irritating – the movement is not irritating (IRRITATING); (9) the movement is slow – the movement is quick (SLOW); (10) it is difficult to move my fingers – I can move my fingers with ease (DIFFICULTY); and (11) it is impossible to extend the finger sufficiently – it is possible to extend the finger sufficiently (IMPOSSIBLE EXTENSION). The participants responded using a 7-point semantic differential scale (negative items, 7: completely agree, 6: definitely agree, 5: slightly agree, 4: neither agree nor disagree, 3: slightly agree, 2: definitely agree, 1: completely agree, positive items). All negative items on the QMF were created based on the following procedures. Immediately after the individuals with hemiplegia performed hand movements for the movie stimuli, they were asked to freely describe what they were feeling about their hands and movements in their hemiplegic sides. We then confirmed their responses by asking them whether they felt they had answered the items completely or not. We then adopted 11 common “completely felt” items described by the six individuals with hemiplegia, as negative items on the QMF. We then created the positive items to represent the opposite of the negative items. Thus, higher questionnaire scores for the hemiplegic hands were considered to indicate that the participant more accurately understood the performers' feelings. For example, if the participants answered “completely agree” to the negative item “the hand is heavy”, we considered it to reflect that they accurately understood the performer's feeling.

### 2.6 Measurement of empathic ability

After evaluating the observed hand movements via QMF, the participants were asked to complete the IRI (Davis, 1983), which is the most widely used self-administered questionnaire for measuring individuals' dispositional empathy. The IRI comprises four subscales, each measuring a distinct component of empathic traits: (1) perspective-taking (IRI-PT) describes the ability to cognitively adopt another person's psychological point of view; (2) fantasy (IRI-FS) describes spontaneous tendencies to become immersed in the feelings and actions of fictional situations; (3) empathic concern (IRI-EC) describes feelings of emotional compassion and concern for others; and (4) personal distress (IRI-PD) describes “self-focused” negative feelings of personal anxiety and unease in response to others' tense experiences. Previous data indicate that AON and rTPJ activation are modulated by highly empathic traits (Lamm et al., 2010; Moriguchi et al., 2007). We used this measurement scale to clarify whether the AON and rTPJ responses in our experiment were related to the participants' empathic abilities or to their pseudoexperience with hemiplegic movements.

### 2.7 fMRI data analyses

Image preprocessing and statistical analyses were performed using SPM8 (Wellcome Department of Imaging Neuroscience, London, UK), implemented in MATLAB R2014b (Mathworks, Inc., Sherborn, MA, USA). To correct for interscan head movements, EPI images were realigned to the first image, and the slice timing of each image was corrected to the middle slice. Next, the mean of the realigned EPI images was co-registered with the T1-weighted MR images, after which the co-registered T1-weighted images were normalized to the Montreal Neurological Institute (MNI) template. The parameters from this normalization process were applied to each EPI image. Finally, the normalized EPI images were spatially smoothed using a Gaussian kernel of 8 mm full-width at half-maximum.

A first-level participant-wise analysis was performed using a general linear model (GLM) with the hemodynamic response function modelled as a boxcar function. The realignment parameters were included in the GLM as confounding covariates. For each participant, we generated statistical parametric maps of the *t* statistic and stored the contrast images for a second-level random effects analysis to enable population inferences. For within-group analyses, the main effects for observing hemiplegic hand movements (i.e. HHM vs. non-HHM and non-HHM vs. HHM) were separately computed for PTs and NPs using one-sample *t-*tests. For between-group analyses of contrast images, we used a two-sample *t*-test to compare brain activation between PTs and NPs using contrast images of the main effects from each group (i.e. HHM vs. non- HHM and non-HHM vs. HHM). For all comparisons in the second-level analyses, the voxel-level threshold was set to *P* < .001 (uncorrected), and the cluster-level threshold was set to *P* < .05 with correction for family-wise error (FWE). We then extracted the eigenvariate values (parameter estimates, mean ± standard error) from any activated clusters that showed significant differences in interactive comparisons of [PTs (HHM vs. non-HHM) vs. NPs (HHM vs. non-HHM)].

After we identified the significantly activated cluster in the above interaction contrast, we performed multiple regression analysis to test the effect of the degree of understanding the HHM feeling, the personal empathic trait, and the participants' pseudoexperience factor on the rTPJ activity, which was an activated cluster in the above- interaction contrast. Given a priori evidence that the rTPJ is critically involved in understanding of another person's mental perspective and closely related to an individual's empathic traits, we hypothesized that the rTPJ activity in our experiment would be predicted either by the participants' pseudoexperience factor (PT or NP), or by the degree of understanding of hemiplegic states (i.e., the QMF), rather than empathic trait (i.e., the IRI). Before implementing this analysis, composite variables were generated for each questionnaire's scores, such as the sum scores of 11 items in the QMF and those of four subscales in the IRI for each participant. Thereafter, parameter estimates of the rTPJ were entered as dependent variables and the composite variables of QMF, those of IRI, and the participants' factors (PT or NP) were entered as independent variables. The level of significance was set at *P* < .05.

### 2.8 Effective connectivity analyses

We performed psychophysiological interaction (PPI) analyses, which assessed the hypothesis that activity in a brain region can be explained by an interaction between a cognitive process and the activity in another region, to identify brain regions that showed stronger covariation with the rTPJ during PT observations of HHMs (Friston et al., 1997). The results of our second-level analyses for the comparison [PTs (HHM vs. non-HHM) vs. NPs (HHM vs. non-HHM)] revealed greater activation in the rTPJ; therefore, we designated the rTPJ as the seed region. Notably, as mentioned above, the rTPJ is critically involved in assuming another person's mental perspective and is functionally linked with the AON (Brass and Heyes, 2005).

Individual volumes of interest (VOIs) were generated as spheres with a 3-mm radius around local maxima for the rTPJ. The sphere center was determined based on the coordinates of brain regions that were significantly activated in the second-level analyses comparing [PTs (HHM vs. non-HHM) vs. NPs (HHM vs. non-HHM)] (rTPJ: *x* = 52, *y* = −32, *z* = 20). We aimed to set the radius size so as not to deviate from the activated rTPJ cluster, and it was also based on previous studies implementing PPI analyses (Decety et al., 2008; Decety and Porges, 2011; Yoder et al., 2015). PPI analyses consists of three regressors: the psychological variable, the physiological variable, and the PPI term (i.e., the interaction between two variables). For the physiological variable, we first estimated the actual neural activity in the VOI (i.e. rTPJ) by extracting the time series for each PT and NP. This activity within the rTPJ was used as the physiological variable. Next, the psychological variable was vector coded for the effects of the PTs' pseudoexperience with hemiplegia (1 for HHM, −1 for non-HHM) and that of the NPs' same contrast (1 for HHM, −1 for non-HHM), which were convolved with hemodynamic response function (HRF). Finally, to create PPI regressors, the deconvolved physiological variables were multiplied by the psychological vectors and then reconvolved with HRF. Through these processes, participant-wise PPI models were created, and contrast images were generated for positive PPIs. These contrast images were entered into a second-level analysis for contrasts of interest. Clusters showing differing connectivity between the chosen conditions were visualized using SPM *t*-maps. The voxel-level threshold was set to *P* < .001 (uncorrected), and the cluster-level threshold was set to *P* < .05 (corrected for FWE).

### 2.9 Behavioral data analysis

For behavioral analyses, the main dependent variables were the subjective rating scores for each QMF item. Mann-Whitney *U* tests were performed to compare the results between PTs-HHM vs. NPs-HHM and between PTs-non-HHM vs. NPs-non-HHM. Wilcoxon signed-rank tests were used for comparisons between PTs-HHM vs. PTs-non-HHM and between NPs-HHM vs. NPs-non-HHM. Bonferroni correction was performed to avoid type I error. For each test, the level of significance was set at *P* < .0125 (i.e., 0.05 was divided by four, the number of above tests). For analyses of IRI scores in total and for each subscale, Mann-Whitney U tests were performed to compare scores between PTs and NPs. For each test, the level of significance was set at *P* < .05. Thereafter, to clarify the effect of an individual's empathic traits on the ability to understand the feeling states of hemiplegic movements, Spearman's rank correlation analyses were performed on each IRI score (IRI-PT, IRI-FS, IRI-EC, and IRI-PD) with the scores of each item in the QMF calculated for both PTs and NPs. To avoid type I error, we performed Bonferroni correction. For each correlation test, the level of significance was set at P < .0125 (i.e., 0.05 was divided by four, the number of correlation tests based on the four IRI subscales). Behavioral data analyses were performed using SPSS statistical software version 18.0 (SPSS Statistics Base; IBM, Inc., Chicago, IL, USA).

## 3 Results

### 3.1 QMF scores

**Fig. 2:**
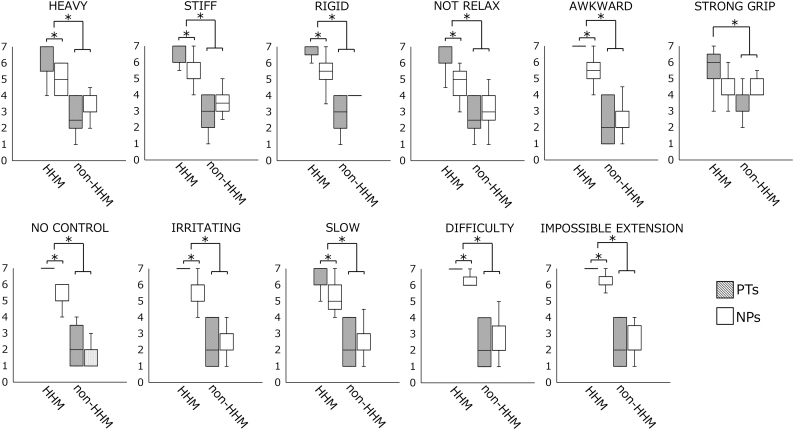
Box plots depicting the upper quartile (75%), median (50%), and lower quartile (25%) of the subjective rating scores for each questionnaire item. PTs: physical therapists; NPs: naïve participants; HHM: hemiplegic hand movements; non-HHM: non-hemiplegic hand movements.

Fig. 2 shows the upper quartile (75%), median (50%), and lower quartile (25%) of the subjective rating scores for each QMF item. Compared to the NPs, PTs provided significantly higher median ratings for all items in the HHM condition (*P* < .001 for all differences). In the non-HHM condition, the median ratings did not significantly differ between the PT and NP groups, with the exception of the STRONG GRIP item.

### 3.2 IRI scores

The median IRI total score and subscale scores did not significantly differ between the PT and NP groups: Total-IRI (PT = 79, NP = 78; *P* = .95), PT-IRI (PT = 20, NP = 22; *P* = .80), FS-IRI (PT = 20, NP = 21; *P* = .73), EC-IRI (PT = 21, NP = 22; *P* = .60), PD-IRI (PT = 17, NP = 18; *P* = .67).

### 3.3 Correlation results between QMF and IRI scores

With regard to the PTs, we did not observe any significant correlations between each IRI score (IRI-PT, IRI-FS, IRI-EC, and IRI-PD) and the scores for each QMF item. With regard to the NPs, we observed a significant positive correlation between the scores only for IRI-PT and those for four QMF negative items (STIFF: *r* = 0.79, *P* < .0125; NOT RELAXED: *r* = 0.68, P < .0125; NO CONTROL: *r* = 0.82, P < .0125; and IRRITATING: *r* = 0.63, P < .0125). On the other hand, none of the NPs' scores for IRI-EC, IRI-FS, or IRI-EC significantly correlated with the scores for each QMF item.

### 3.4 Brain imaging

**Fig. 3:**
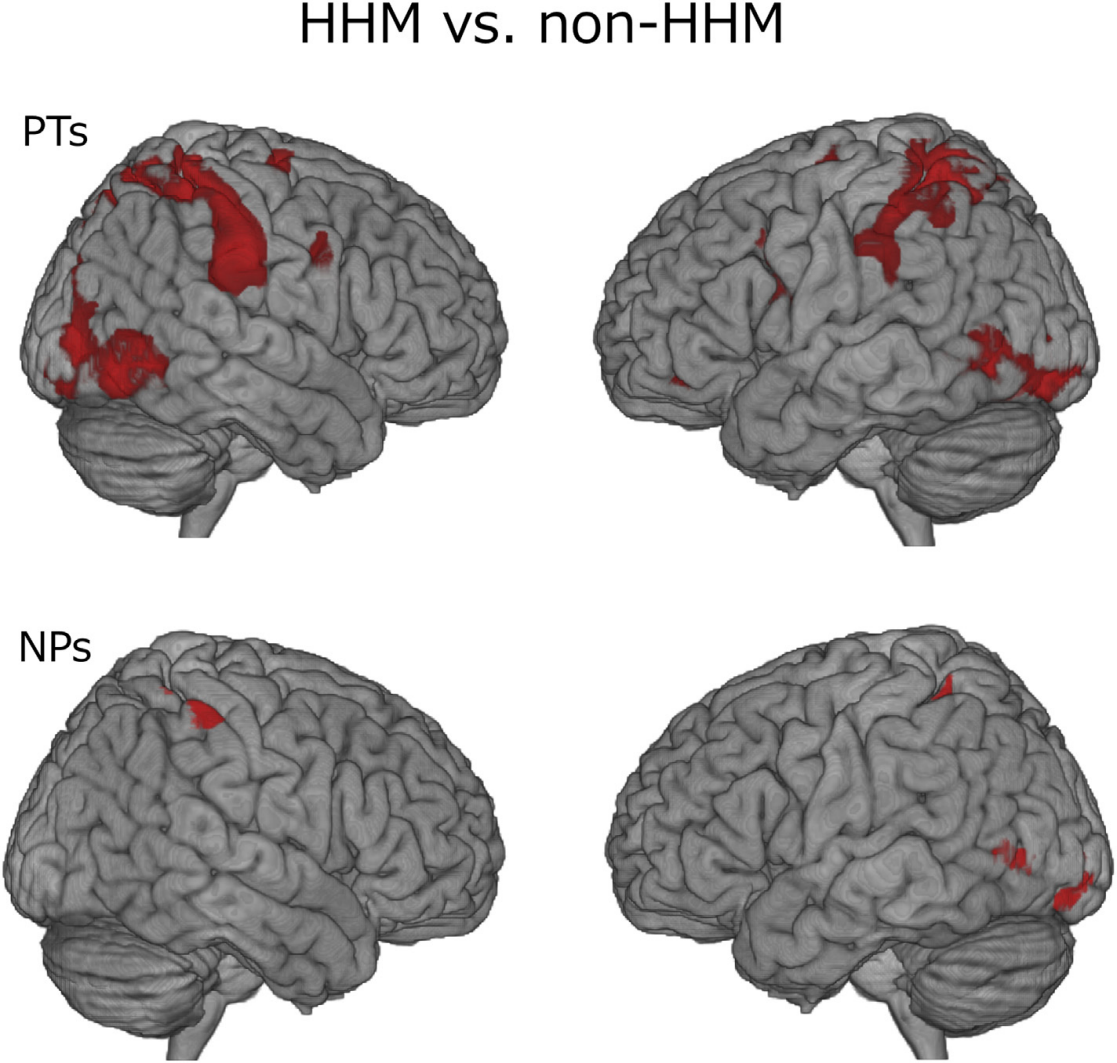
Brain regions exhibiting significant activation for the contrast of HHM vs. non-HHM. Top: The PT group. Bottom: The NP group. PTs: physical therapists; NPs: naïve participants; HHM: hemiplegic hand movements; non-HHM: non-hemiplegic hand movements.

**Fig. 4:**
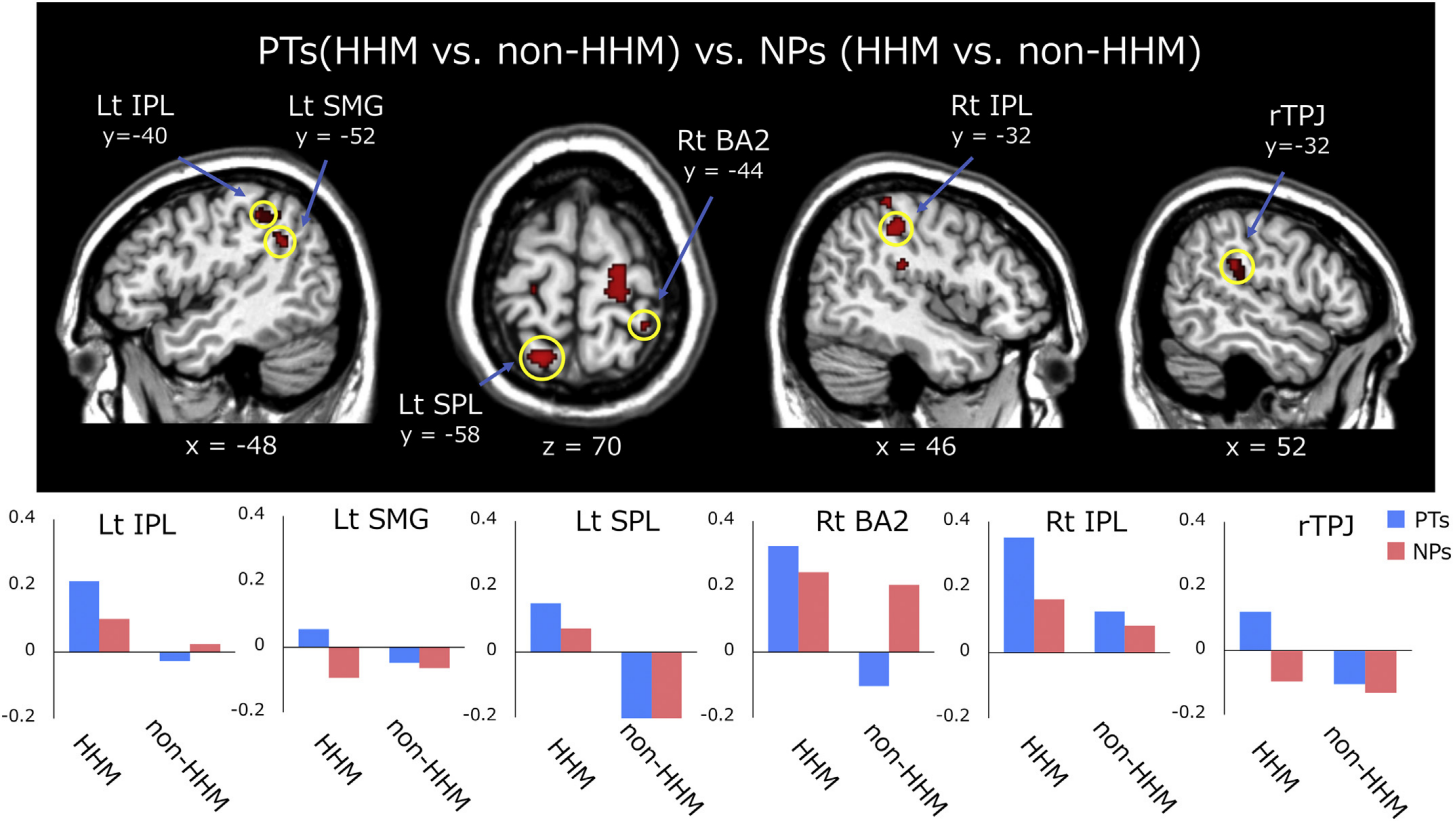
Brain regions exhibiting significant activation for the contrast [PTs (HHM vs. non-HHM) vs. NPs (HHM vs. non-HHM)], and the parameter estimates from the activated clusters under each of the following conditions: PTs-HHM, NPs-HHM, PTs-non-HHM, and NPs-non-HHM. Rt: right; Lt: left; IPL: inferior parietal lobule; SMG: supramarginal gyrus; SPL: superior parietal lobule; BA2: Brodmann area 2; rTPJ: right temporoparietal junction; PTs: physical therapists; NPs: naïve participants; HHM: hemiplegic hand movements; non-HHM: non-hemiplegic hand movements.

Fig. 3, Fig. 4 and Table 1, Table 2 present the regions of significant neural activation revealed by second-level random-effects analyses. We compared the HHM and non-HHM conditions within each group. In the HHM condition, when compared to the non-HHM condition, the PT group exhibited significantly greater brain activation in the main components of the AON, encompassing the frontoparietal and temporal areas and several visual areas (Table 1). The opposite contrast (non-HHM vs. HHM) for the PT group revealed significant activation in several primary visual areas, but none within the AON.

**Table 1:** Brain regions with significant activation under the (HHM – non-HHM) contrast.

L/R	Region	MNI coordinate			t
		x	y	z	
The PT group					
R	Inferior parietal lobule	46	−28	42	7.40
L	Inferior parietal lobule	−40	−40	50	5.80
R	Superior parietal lobule	20	−62	60	9.01
L	Superior parietal lobule	−34	−54	64	7.57
L	Ventral premotor area	−52	6	22	4.82
R	Ventral premotor area	50	0	36	4.64
R	Dorsal premotor area	24	−10	56	6.50
R	Dorsal premotor area	−24	−10	64	5.55
L	Inferior frontal gyrus	−42	40	−14	4.91
R	Somatosensory area (BA1, 2)	46	−28	42	7.83
L	Somatosensory area (BA1, 2)	−40	−44	62	6.39
L	Middle temporal gyrus (V5/MT)	−48	−64	5	4.24
L	Inferior occipital gyrus (V4)	−28	−96	−6	8.14
L	Middle occipital gyrus	−34	−72	16	4.95
R	Inferior temporal gyrus	48	−62	−8	6.95
R	Middle temporal gyrus (V5/MT)	52	−60	−2	7.06
R	Inferior occipital gyrus (V4)	36	−88	−4	7.64

The NP group					
R	Inferior parietal lobule	60	−3	48	4.69
L	Superior parietal lobule	−42	−46	64	4.31
R	Inferior occipital gyrus (V3)	30	−92	−8	6.27
L	Middle occipital gyrus (V4)	−30	−96	0	5.37
L	Middle occipital gyrus (V4)	−52	−74	0	5.18

The threshold was set was set at voxel level of *p* < .001 (uncorrected) and cluster level of *p* < .05 (family wise error (FEW) corrected). PTs: physical therapists; NPs: naïve participants; HHM: hemiplegic hand movements; non-HHM: non-hemiplegic hand movements.

**Table 2:** Brain regions with significant activation under the [PTs (HHM vs. non-HHM) vs. NPs (HHM vs. non-HHM)] contrast.

L/R	Region	MNI coordinate			t
		x	y	z	
R	Dorsal premotor area	22	−22	66	4.10
R	Right temporoparietal junction	52	−32	20	3.43
R	Inferior parietal lobule	46	−32	46	3.92
L	Inferior parietal lobule	−44	−40	46	3.77
L	Supramarginal gyrus	−48	−52	30	3.71
L	Superior parietal lobule	−20	−58	70	4.17
R	Primary somatosensory area(BA2)	40	−44	64	4.54
L	Primary motor area	−22	−26	68	3.73
L	Middle cingulate cortex	−16	2	44	4.09

The threshold was set was set at voxel level of p < .001 (uncorrected) and cluster level of *p* < .05 (family wise error (FEW) corrected). PTs: physical therapists; NPs: naïve participants; HHM: hemiplegic hand movements; non-HHM: non-hemiplegic hand movements.

Within the NP group, we detected significantly stronger brain activation for the HHM condition than for the non-HHM condition within several visual areas and in a parietal portion of the AON. The opposite contrast (non-HHM vs. HHM) revealed greater activation only in several primary visual areas.

To investigate the effects of interactions related to the participant group and HHM observation, we calculated the differential activity associated with these two factors. We calculated the contrast [PTs (HHM vs. non-HHM) vs. NPs (HHM vs. non-HHM)] to identify regions in which the effect of HHM observation was associated with greater neural response in the PT group than in the NP group. The results revealed significant activation in several components within the AON and other regions, including the right dorsal premotor area (PMd), bilateral inferior parietal lobule (IPL), left supramarginal gyrus (SMG), left superior parietal lobule (SPL), right primary somatosensory area (BA2), rTPJ, left middle cingulate cortex, and right superior temporal gyrus (Fig. 4 and Table 2). The opposite contrast [NPs (HHM vs. non-HHM) vs. PTs (HHM vs. non-HHM)] did not reveal significant activation in any regions.

In the multiple regression analysis, the model including the participants' pseudoexperience factor, QMF, and IRI was statistically significant [adjusted R^2^ = 0.22, *P* < .01]. However, none of the independent variables in the model were significant [the participants' pseudoexperience factor; β = 0.49, *t* = 1.65, *P* = .11; the QMF; β = 0.04, *t* = 0.13, *P* = .9; and the IRI; β = 0.11, *t* = 0.5, *P* = .5].

### 3.5 Psychophysiological interaction (PPI) analyses

**Fig. 5:**
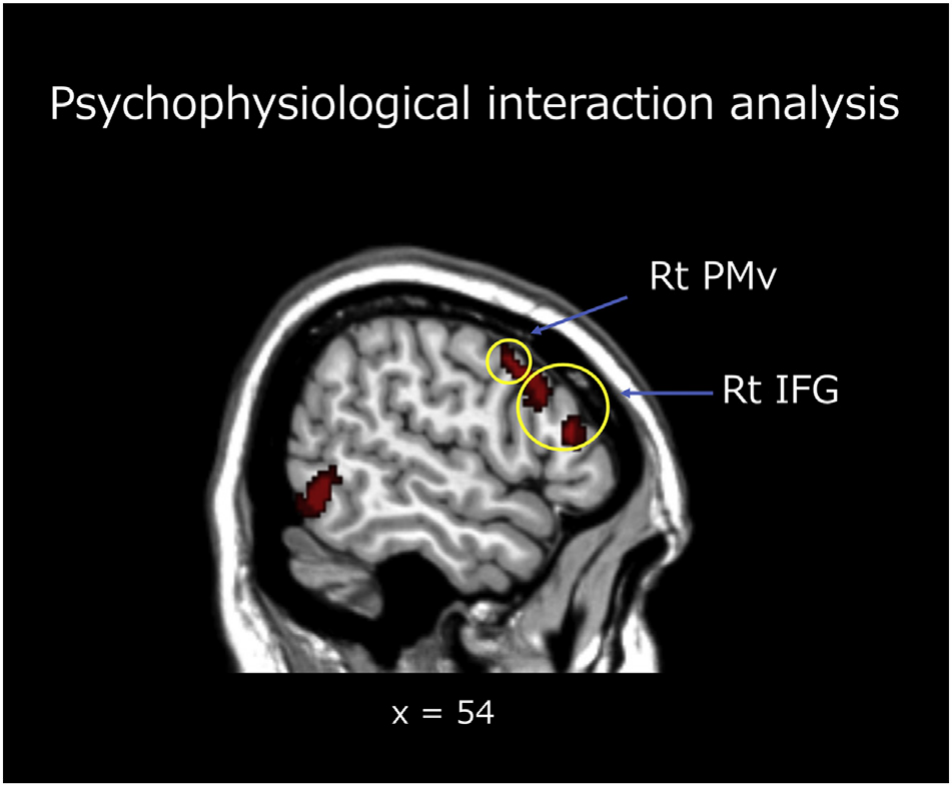
Brain regions exhibiting increased effective connectivity with the rTPJ during physical therapists' observations of hemiplegic hand movements. Rt: right; PMv: ventral premotor area; IFG: inferior frontal gyrus.

We further aimed to clarify the neural mechanisms associated with understanding the mental states of individuals with hemiplegic movements and to assess possible differences in these mechanisms between PTs and NPs. To this end, we performed PPI analysis using the rTPJ as the seed region in each group. When PTs observed HHMs, we observed increased effective connectivity between the rTPJ and some areas of the AON (the right IFG, right PMv, and right PMd), bilateral visual areas, and the cerebellum (Fig. 5 and Table 3). However, we failed to find increased connectivity between the rTPJ and any other areas while the NPs observed HHMs.

**Table 3:** Psychophysiological interaction analysis.

L/R	Region	MNI coordinate			t
		x	y	z	
R	Inferior frontal gyrus	44	18	26	5.33
R	Inferior frontal gyrus	52	32	18	5.46
R	Ventral premotor area	54	10	38	4.23
R	Dorsal premotor area	42	0	54	3.93
R	Fusiform gyrus	32	−68	−8	5.75
L	Inferior occipital gyrus	−44	−88	−4	5.19
L	Middle occipital gyrus	−18	−100	6	5.09
L	Middle occipital gyrus	−60	−54	−4	4.78
L	Cerebellum	−12	−74	−46	6.23
L	Cerebellum	−38	−54	−44	4.64
R	Cerebellum	28	−60	−46	5.00

Analysis for the rTPJ × PTs during observation of hemiplegic hand movements. The threshold was set was set at voxel level of *p* < .001 (uncorrected) and cluster level of *p* < .05 (family wise error(FEW) corrected). rTPJ: right temporoparietal junction; PT: physical therapist.

## 4 Discussion

In the present study, we investigated whether the neural mechanisms associated with understanding the bodily and mental states of individuals with hemiplegic movements differed between PTs who had pseudoexperience with hemiplegia during clinical practice versus NPs. The PTs who viewed hemiplegic movements exhibited increased activation in some components of the AON and rTPJ when compared to that observed when the NPs viewed the same movements. Furthermore, PPI analyses revealed increased connectivity between the rTPJ and some components of the AON during the PTs' observation of HHMs. In the HHM condition, QMF ratings of all items were significantly higher in the PT group than in the NP group. Moreover, the IRI total and subscale scores did not significantly differ between PTs and NPs, suggesting that PTs did not have a greater general empathic ability than NPs. Overall, our findings suggest that, pseudoexperience with hemiplegic movements in highly-experienced PTs enhances the recruitment of the AON and rTPJ in these individuals when they observe hemiplegic hand movements, enabling them to more accurately understand hemiplegia-associated physical feelings and mental states.

### 4.1 Effects of pseudoexperience with hemiplegic movements on the understanding of bodily states

The interaction contrast [PTs (HHM vs. non-HHM) vs. NPs (HHM vs. non-HHM)], reflecting the effect of HHM observation in the PT group relative to the NP group, revealed greater activation in the bilateral IPL, left SMG, left SPL, and right BA2. These areas are considered main components of the AON, which is prominently involved in action understanding (Calvo-Merino et al., 2006; Cross et al., 2009; Gazzola and Keysers, 2009; Iacoboni, 2005; Turella et al., 2013). Indeed, previous data indicate that the IPL—especially the SMG—plays a key role in bodily and movement representation (Calvo-Merino et al., 2006; Naito et al., 2005; Rushworth et al., 2001). The SPL is involved in maintaining and updating the representation of bodily posture (Felician et al., 2004; Parkinson et al., 2010). Furthermore, the BA2 is responsive to afferent proprioceptive information and is reciprocally connected to the IPL (Keysers et al., 2010). Their functions suggest that these parietal areas likely underlie a person's ability to understand the physical feelings associated with observed movements. Our present findings suggest that the clinical experience of PTs, such as careful handling and observation, may update their bodily representations to include hemiplegic bodies and movement states, allowing them to accurately understand the physical states associated with hemiplegia.

The PTs exhibited greater AON activation for HHMs, but they should have been more familiar with non-HHMs, as none had directly experienced hemiplegia with their own hands. Similar to the PTs, the NPs exhibited more robust activation in some regions of the AON for HHMs than for the non-HHMs. One possible explanation for these results is that AON activation varies with the physical effort and complexity of the observed actions. Indeed, QMF scores revealed that both the PTs and NPs appraised feelings as more effortful or complex in the HHM condition compared to the non-HHM condition, in agreement with previous imaging data regarding action observation (Alaerts et al., 2010; Biagi et al., 2010). Biagi et al. (2010) reported greater activation in certain AON components (e.g. the parietal area) when participants observed complex finger movements compared to simple finger movements. Importantly, compared to the NPs, the PTs in our study exhibited greater increases in AON activity for the HHM condition and provided higher subjective QMF ratings. This suggests that the PTs' pseudoexperience with hemiplegia enabled them to more accurately recognize hemiplegic physical feelings.

However, both groups (PTs and NPs) showed greater activation in only several primary visual areas in the non-HHM condition when compared to the HHM condition. In accordance with the above notion, one might speculate that this condition appears so simple that it is not necessary to produce AON activation. Instead, the participants simply analyze the movement visually through the visual areas. This finding also supports the notion that PTs exploit their bodily representation for accurate assessment of HHM via the AON activation.

### 4.2 Effects of pseudoexperience with hemiplegic movements on inferences regarding mental states

The interaction contrast [PTs (HHM vs. non-HHM) vs. NPs (HHM vs. non-HHM)] revealed increased activation in the rTPJ. Moreover, compared to the NPs, PTs provided higher subjective ratings for emotional items in the QMF during the HHM condition (i.e. NO CONTROL, DIFFICULTY, and IRRITATING), reflecting their nearly complete agreement of hemiplegia-associated mental states (see box plots of these items in Fig. 2). Accordingly, previous studies suggest that the rTPJ is involved in the perception of another person's affective mental states (Decety and Lamm, 2007; Mitchell, 2009; Saxe, 2006; Saxe and Kanwisher, 2003). The multiple regression analysis showed that the none of variables could predict the rTPJ activity. One explanation for this finding is either multicollinearity among the dependent variables, or the small sample sizes used for this analysis. In this study, however, we consider that direct comparisons of each variable between groups, will better emphasize and, directly address the effect of pseudoexperience. Overall, our results suggest that PTs' pseudoexperience with hemiplegia exerts effects to exhaustively understand the negative mental states associated with hemiplegia.

However, some previous studies reported that the rTPJ plays a role in visuospatial transformation, such as adopting the spatial perspective of an observed other's action by imaging observers themselves in the perspective of the other (Arzy et al., 2006; Blanke et al., 2005). However, in the present study, all of the hand stimuli were presented from the same third person perspective so that our participants might process all of the stimuli in a similar way, regardless of the stimulus condition. On the other hand, the most robust activation in the rTPJ occurred when the PTs observed HHMs with ratings that were the most accurate in some negative items. It seems to reflect PTs' processes to reason the more complicated feeling states associated with HHMs (i.e. NO CONTROL, DIFFICULTY, and IRRITATING) rather than the process of visuospatial transformation of HHMs.

When the PTs viewed HHMs, we also observed an increased effective connectivity between the rTPJ and portions of the AON, including the IFG, PMv, and PMd. This finding is in accordance with previous data suggesting a close relationship between the MNS and the rTPJ during mentalization regarding other people's mental states (Brass et al., 2009; Decety and Grezes, 2006; Lombardo et al., 2010). Lombardo et al. (2010) demonstrated that when participants attempt to infer the mental state of themselves or others, neural signals in the rTPJ interact with those in portions of the MNS (i.e. the PMv, PMd, and IFG). When observing another person's action, the MNS may first mirror the action to develop a shared representation. Subsequently, the mentalizing system of the rTPJ is activated, enabling the observer to assume the mind of the other person (Brass et al., 2009). Together with the behavioral findings that PTs rated almost complete agreement with the mental-related items, it is conceivable that their bodily-related neural system, via the rTPJ, greatly contributes to the exhaustive understanding of the mental-states associated with hemiplegic movements—even though they are not a clinical mental health care provider, such as a clinical psychologist or a psychiatric occupational therapist. PTs may map the difficulty with movement based on their own bodily representation, and then apply this difficulty associated information to appropriately infer the mental states associated with a particular movement. This kind of exhaustive mentalizing ability, based on observing movements in persons with disability, may be the highly pseudoexperienced PT's specific skill.

Our present findings do not indicate which aspect of pseudoexperience (e.g. observation of or physical contact with patients with hemiplegia) exerts a greater influence on the observer's understanding. Future studies are required to further elucidate the effects of pseudoexperience by examining the direct influence of each experiential factor; for example, an interventional study could be performed with participants who have experience with direct touching or only observation of individuals with hemiplegia. In addition, another future study in which individuals with hemiplegia are recruited as study participants are also required to investigate whether the effect of pseudoexperience for accurate understanding the hemiplegic states is similar to that of actual experience with hemiplegia.

### 4.3 The effect of empathic traits on the understanding of hemiplegic states

The behavioral results demonstrated that there is a significant positive correlation between the scores for perspective-taking ability (i.e., IRI-PT) and the subjective ratings for some QMF items, only in the NPs and not in the PTs. These findings suggest that NPs who have greater ability to adopt another person's psychological point of view are capable of better identifying the physical and mental states of hemiplegia to some extent. In particular, the NPs' perspective-taking ability is strongly related to the degree of understanding the somatosensorial and mental states associated with hemiplegia (i.e., the items: STIFF, NOT RELAXED, NO CONTROL, and IRRITATING). This is consistent with previous notions that adopting the perspective of another person induces a strong empathic response (Lamm et al., 2007; Myers et al., 2013). Thus, perspective-taking ability of the NPs would play a role in understanding the difficulties experienced by hemiplegic individuals.

In contrast to the NPs' correlation results, the PTs' IRI scores were not related to the scores for any of the QMF items, suggesting that their understanding may rely upon their accumulated pseudoexperience of hemiplegia based on their clinical treatment session or practice rather than perspective-taking skills or other empathic traits. Thus, their pseudoexperience appears to function critically in the comprehension of hemiplegic states when taken with behavioral results, especially given that their ratings for all the QMF items were higher than those of the NPs.

### 4.4 Implication for clinical care

Given our behavioral findings, accumulating pseudoexperience with hemiplegia through clinically touching and observing patients with hemiplegia is considered to be preferentially responsible for an accurate understanding and assessment of patients with hemiplegia. Such an accumulating pseudoexperience process is considered to be more significant than heightening inherent empathic traits for the appropriate understanding of patients' states. Thus, even if they are not highly empathic therapists, accumulating experience through their own sensory systems such as touching and observing their patients would enhance their clinical skills.

Furthermore, this assessment should be instantly processed in a PT's brain because the AON is thought to intuitively map observed movements on observers' bodily representations (Gallese and Goldman, 1998; Keysers and Gazzola, 2007). Considering that accurate and instant assessment is the most fundamental process in physical therapy, such assessments based on pseudoexperience may enable PTs to administer more comprehensive treatments to their patients. It is conceivable that constant accumulation of pseudoexperience with hemiplegia in routine clinical practice or training should improve PTs' skills for better rehabilitation treatment.

In addition, the effect of clinical pseudoexperience probably extends to a PT's daily life. For example, PTs may instantaneously be more able to notice the suffering of individuals with disabilities on the street, trains, and elsewhere, enabling them to provide an assistance for them. Future investigations are needed to test whether accumulated pseudoexperience can exert clear influences on outcomes of treatments by comparing the clinical skills of highly-trained PTs and those of PTs with little experience by measuring brain activity.

## 5 Conclusion

In the present study, our findings suggest that the PTs' pseudoexperience enabled them to conduct a more thorough observational assessment of the physical and mental states of individuals with hemiplegia. Furthermore, the PTs' ability, related to action understanding, would contribute to a more critical assessment of the mental states associated with hemiplegic movements. Thus PTs with significant pseudoexperience may possibly offer more comprehensive physical therapy treatment for their clients.

## Conflicts of interest

The authors declare that they have no conflicts of interest.

## Funding

This work was supported by a Grant-in-Aid from JSPS (Japan Society for the Promotion of Science) fellows [grant number 201505325].
